# Amniotic membrane transplantation ineffective as additional therapy in patients with aggressive Mooren’s ulcer

**DOI:** 10.1186/1471-2415-13-81

**Published:** 2013-12-17

**Authors:** Maurice Schallenberg, Henrike Westekemper, Klaus-Peter Steuhl, Daniel Meller

**Affiliations:** 1 Department of Ophthalmology, University Hospital Essen, University of Duisburg-Essen, Hufelandstr 55, D-45147 Essen, Germany; 2 Augenklinik, HELIOS Klinikum Wuppertal, Heusnerstrasse 40, D-42283 Wuppertal, Germany

**Keywords:** Cornea, Mooren’s ulcer, Amniotic membrane, Autoimmune disease, Immunosuppressive therapy

## Abstract

**Background:**

Mooren’s ulcer is a severe ulcerative inflammation of the cornea. The exact pathogenesis remains unclear. Therefore many therapies of Mooren’s ulcer are recommended in literature. To shed more light on the ongoing question of optimal treatment of severe progressive Mooren’s ulcer, we here report on a retrospective case series of patients treated with systemic immunosuppressive therapy and additional amniotic membrane transplantation.

**Methods:**

Medical records from seven patients (eleven eyes), 4 male and 3 female, with severe progressive Mooren’s ulcer were analysed retrospectively. The mean follow up was 88.4 ± 80.8 months (range 12–232 month). A HLA-typing was performed in all patients. A systemic immunosuppressive therapy was administered in all patients. The amniotic membrane was transplanted after the base of the ulcer was resected.

**Results:**

Multiple amniotic membrane transplantations were necessary in six patients. The visual outcome of all patients was poor. No patient achieved a visual acuity better than 20/630 Snellen chart. Five patients were positive for HLA-DQ2 and four patients were positive for HLA-DR17(3).

**Conclusions:**

The aggressive and highly inflammatory form of Mooren’s ulcer is difficult to treat and the progression of the disease is hard to influence positively even under systemic immunosuppressive therapy. Therefore, the main intention of therapy is to achieve a stable epithelialized corneal surface without the risk of perforation. Amniotic membrane transplantation is not able to cure severe forms of Mooren’s ulcer. However it supports the immunosuppressive therapy in acute situations as in critical corneal thinning.

## Background

Mooren’s ulcer is a severe ulcerative, uni- or bilateral inflammation of the cornea. Typically the inflammation starts in the peripheral cornea and the ulcer enlarges centrally and circumferentially – commonly associated with severe ocular pain. In late stages of the disease the destruction of the peripheral corneal stroma results in a conjunctivalized descemet membrane and in some cases corneal perforations occur. The progression and outcome of Mooren’s ulcer differ between patients though the clinical appearance is similar. The exact pathogenesis remains unclear even though the disease was first described by Bowman more than 150 years ago [1]. It is consensus that the Mooren’s ulcer is an autoimmune disease that targets the cornea without other systemic disease association. Support for this idea came from Gottsch and colleagues who found antibodies against Calgranulin C in the serum of patients with Mooren’s ulcer [2,3]. Calgranulin C is uniquely expressed in cornea stromal tissue. Epidemiological studies from India identified a history of ocular trauma, previous cataract surgery, bacterial and helminth infection as risk factors to develop Mooren’s ulcer [4].

Furthermore the disease occurs in populations living abroad and second generation migrants. Therefore the question of genetic predisposition arises. The highly polymorphic human lymphocyte antigens play an important role in immune response. An association to several autoimmune diseases such as rheumatoid arthritis, Graves’disease, and multiple sclerosis has been described [5]. Taylor and coworkers identified an association of HLA-DR17(3) and HLA-DQ2 to Mooren’s ulceration [6].

Various therapies of Mooren’s ulcer are recommended in literature. The excision of the conjunctiva around the ulceration combined with local or systemic steroids or coagulation of the base of the ulceration showed no long term benefit [7,8]. Authors from a large Chinese study of 550 patients who were treated with lamellar keratoplasty after topical resection of the ulceration, concluded that an adjuvant medication of topical ciclosporin A 1% eye drops improves outcome [9]. They recommended topical ciclosporin A as therapeutic approach to modulate immune response. The stepladder of immunosuppressive agents used in Mooren’s ulcer include prednisolone, methotrexate, azathioprine, cyclosporine, cyclophosphamide, and infliximab [9-12].

Recent studies suggest amniotic membrane transplantation (AMT) as a therapeutic approach in Mooren’s ulcer. The idea of amniotic membrane transplantation as a therapeutic option in Mooren’s ulcer came from the positive results shown by AMT of corneal ulceration due to other reasons. However, results reported in the literature with this procedure vary widely.

Recently, a retrospective study of 18 eyes reported a stabilization of the visual acuity and rapid healing of the epithelial defect after single AMT in most cases [13]. In contrast, results of AMT combined with conjunctival autografting or lamellar keratoplasty are less convincing [14,15].

The growing evidence that Mooren’s ulceration has an autoimmune genesis suggests an immunosuppressive therapy in Mooren’s ulceration. In addition to systemic steroid therapy, ciclosporin A or cyclophosphamide is recommended in cases with severe progression or relapse [16,17].

To shed more light on the ongoing question of optimal treatment of severe progressive Mooren’s ulcer, we here report a retrospective case series of patients treated with systemic immunosuppressive therapy and additional AMT.

## Methods

Medical records from patients with severe progressive Mooren’s ulcer were analysed retrospectively. They were treated between October 2002 and April 2012 at the University Eye Hospital Essen, Essen, Germany. The study was approved by the local ethics committee (Ethics Committee of the University Duisburg-Essen Nr. 13-5501-BO) and adhered to the tenets of the Declaration of Helsinki and local law.

Mooren’s ulcer was diagnosed if an idiopathic painful peripheral corneal ulceration with typical clinical features and absence of scleral inflammation was observed. Infection was excluded by clinical and microbiological investigations. Other diagnoses causing peripheral corneal ulcerations (e.g. rheumatoid arthritis, Wegner’s granulomatosis, other collagen vascular diseases, inflammatory bowel diseases, rosacea, Terrien’s degeneration, or pellucid degeneration) were excluded by interdisciplinary clinical assessments and laboratory investigations. A HLA-typing was performed in all patients.

The systemic immunosuppressive therapy was administered in close collaboration with the clinic for hematology and clinic for nephrology of the University Hospital Essen, Essen, Germany to monitor the potentially serious side effects and the drug levels.

In cases of severe corneal thinning and highly inflammatory ulceration an AMT was performed. The amniotic membrane was obtained from the Cornea Bank Essen. The method of amniotic membrane preparation has been previous described by Kim and Tseng [18]. The surgical procedure was performed under peribulbar anaesthesia. The base of the ulcer was resected and an amniotic membrane graft (basement membrane oriented up) of similar size and shape was laid into the defect and sutured with 10–0 nylon sutures. In some cases the procedure was combined with a conjunctival resection and / or amniotic membrane patch which was sutured with 8–0 vicryl sutures to the peribulbar conjunctiva.

The clinical findings and therapeutic response of AMT in addition to local and systemic immunosuppressive therapy were determined.

## Results

Eleven eyes of seven patients, 4 male and 3 female, were enrolled in this retrospective interventional cases series. The mean age at the first presentation in our clinic was 62.8 ± 15.7 years (range 37–81 years). The mean follow up was 88.4 ± 80.8 months (range 12 – 232 month). Details of the patients’ characteristics and clinical features are summarized in Table 1. One patient had a severe aggressive form of Mooren’s ulcer on both eyes. In all other cases, one eye was affected by severe aggressive Mooren’s ulcer with large deep ulcerations, the other eye was not affected in three cases, and only mild affected in the other three cases, respectively. Five patients were treated with intravenous cyclophosphamide as pulsetherapy and 2 patients with ciclosporin A orally as continuous therapy under drug monitoring. In addition, the patients were treated with ciclosporin A 0.05% eye drops three times daily as local immunosuppressive therapy. The supportive local therapy consisted of prednisolone acetate 1% three times daily, and sodium hyaluronate 0.1% and dexpanthenol, each instilled six times a day. Furthermore autologous serum 20% eye drops were applied six times a day.

**Table 1:** Clinical features of patients with Mooren’s ulceration

**Case No.**	**Eye**	**Age**	**HLA DQ2 / DR17(3)**	**Depth of ulcer**
1	both	81	+/+	2/3
2	OD	68	+/-	2/3
3	OD	68	-/-	2/3
4	OD	37	-/+	2/3
5	both	70	+/+	2/3
6	both	45	+/+	Perforation
7	both	71	+/-	2/3

Five patients were positive for HLA-DQ2 and four patients were positive for HLA-DR17(3).

**Figure 1:**
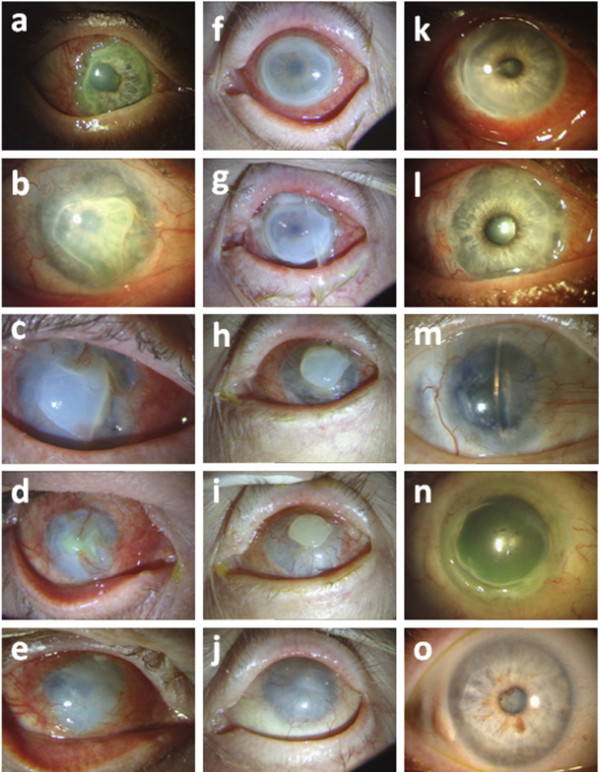
**Representative images of the processes in some patients. a)** - **e)** Right eye of case No. 5: **a)** corneal ulcer at the first visit; **b)** progression of the ulceration; **c)** + **d)** follow up after AMT; **e)** opacified epithelialized cornea at the last visit. **f)** – **j)** Left eye of case No. 1: **f)** peripheral corneal ulcer at the first visit; **g)** progression of the ulceration; **h)** + **i)** follow up after AMT; **j)** opacified epithelilized cornea at the last visit. **k)** – **m)** Right eye of case No.7: **k)** corneal ulcer at the first visit; **l)** progression of the ulceration; **m)** stabilized cloudy cornea with peripheral pannus after AMT and with immunosuppressive therapy. **n)** – **o)** Right eye of case No. 2: **n)** 360° peripheral ulcer at the first visit; **o)** stabilized cornea with peripheral opacified scar under immunosuppressive therapy after AMT.

In 6 patients multiple AMTs were necessary because of relapsing ulceration or persistent epithelial defects. A stabilization of the disease was achieved after one amniotic membrane grafting in only one patient. Representative images of the processes are presented in Figure 1.

The visual outcome of all patients was poor. No patient achieved a visual acuity better than 20/630 Snellen chart in the affected eye with severe aggressive Mooren’s ulceration. Details of surgical procedure, follow up, immunosuppressive therapy and visual outcome are summarized in Table 2.

**Table 2:** Follow up and therapy modalities of patients with Mooren’s ulceration

**Case No.**	**Follow up time (month)**	**Numbers of AM- transplantation/Surgical procedures**	**Immunosuppressive therapy**	**Topical immunosuppressivetherapy**	**BCVA at initial visit**	**BCVA at last follow up**	**Epithelialisation (including conjunctivalisation)**
1	112	OD: 2x + KPL	Cyclophosphamide (i.v.; ineffective) FK-506	Cyclosporin A AT 0.5%	OD: < 20/4000	OD: <20/4000	2 month after AMT and effective immunosuppressive therapy
		OS: 3x			OS: 20/4000	OS: 20/4000	
2	75	OD: 1x	Cyclosporin A (orally)	Cyclosporin A AT 0.5%	OD: 20/2000	OD: 20/4000	3 month after AMT and effective immunosuppressive therapy
3	14	OD: 4x	Cylclophosphamide (i.v.)	Cyclosporin A AT 0.5%	OD: 20/80	OD: 20/2000	1 month after AMT and effective immunosuppressive therapy
4	12	OD: 1x	Cyclophosphamide (i.v.)	Cyclosporin A AT 0.5%	OD: 20/32	OD: 20/80	1 month after AMT and effective immunosuppressive therapy
5	144	OD: 7x + conjunctival resection	Cyclophosphamide (i.v.; ineffective) Cyclosporin A (orally; ineffective) Tacrolimus	Cyclosporin A AT 0.5%	OD: 20/32	OD: <20/4000	3 month after AMT and effective immunosuppressive therapy
					OS: 20/20	OS: 20/32	
6	232	OD: 4x + KPL enucleation	Cyclophosphamide (i.v.)	Cyclosporin A AT 0.5%	OD: 20/25	OD: -	3 month after KPL and effective immunosuppressive therapy
					OS: 20/25	OS: 20/40	
7	30	OD: 5x + conjunctival resection	Cyclophosphamide (i.v.) Azathioprine	Cyclosporin A AT 0.5%	OD: 20/320	OD: 20/800	3 month after AMT and effective immunosuppressive therapy
		OS: 3x + conjunctival resection			OS: 20/32	OS: 20/63	

## Discussion

Mooren’s ulceration is a rare ulcerative inflammation of the cornea with quite a variable clinical progression and outcome for the patients. The diagnosis of Mooren’s ulceration is difficult because other diseases causing a peripheral corneal ulceration have to be excluded in advance. Therefore, randomized prospective therapy studies are difficult to conduct and to date, even a recent Cochrane review in 2011 identified no randomized controlled trials for treatment of Mooren’s ulcer [19].

Reviewing the literature, only case reports or retrospective cases series are published recommending therapeutic approaches based on theoretical considerations [3,9,14,16,17]. The definition of success seems to differ in the published literature. While some studies defined “healing” as success others used the “visual acuity” as primary end point [19].

The natural course of Mooren’s ulceration is very different. In addition to the aggressive ulcerative forms, chronically inflammatory forms and spontaneous remissions are described. These facts make it difficult to evaluate and to compare the different recommended therapeutic approaches. Furthermore, the mean follow up after treatment is usually not longer than two years and in some reports no mean follow up is stated [9].

We report on seven Mooren’s ulcer patients in a long-term follow up. The mean follow up in our interventional case series is 88.4 months ± 80.8. To our knowledge this is the longest follow up ever published on patients with Mooren’s ulcer.

Wood and Kaufman classified Mooren’s ulcer into two main forms according to the age of onset, clinical characteristics, and the prognosis [20]. Type 1 is the benign form, generally monolateral with mild to moderate symptoms. This type was believed to affect mostly adults (over 35 years) and responds well to medical treatment and surgery. In contrast, type 2 is the malignant form that occurs with relatively more pain and generally responses poorly to any treatment. This type affects mostly younger patients (younger than 35 years) and a bilateralcondition in up to 75% of the cases that were reported in black patients. The classification of Watson divided the disease into three types based on the clinical appearance: (1) unilateral Mooren’s ulcer, (2) bilateral aggressive Mooren’s ulcer, and (3) bilateral indolent Mooren’s ulcer [10]. With regard to bilaterality, perforation, age of onset, and recurrence rate our data are not in accordance to the classification of Wood and Kaufman. We report on cases of aggressive Mooren’s ulcer in elderly patients (mean age 62.8 years ± 15.6). These findings are supported by Lewallen and Courtright reviewing the literature on 287 cases of Mooren’s ulcer who found a bilateral disease in 43% of older patients [21]. Chen and coworkers published a consecutive cases series of 550 patients obtained similar results, concluding that the bilateral disease is the malignant type of Mooren’s ulcer [9]. Their findings are in contrast to the result of our case series, in which malignant aggressive processes were observed in unilateral Mooren’s ulcer.

HLA-DQ2 and/or HLA-DR17(3) have been suggested to have a positive correlation to Mooren’s ulcer. All of our patients tested positive for HLA-DQ2 or HLA-DR17(3). We believe that the expression of HLA-DQ2 and/or HLA-DR17(3) may serve as prognostic factor in Mooren’s ulceration, and may help to distinguish the severe aggressive form from the mild benign form of the disease. Further studies with larger patient cohorts are required to investigate this suggestion.

The growing evidence of Mooren’s ulceration as an autoimmune disease has led to the recommendation of systemic immunosuppressive therapy in severe progressive, highly inflammative Mooren’s ulcer [22]. Cyclophospamide and ciclosporin A are the most commonly used agents. Cyclophosphamide may be affective by suppressing B lymphocytes, which produce autoantibodies and promote an immune complex reaction [23]. In contrast, ciclosporin A may work by suppression of the T helper cell and stimulation of the T suppressor cell and cytotoxic T cells [24]. Both therapies are able to halt the progression in many patients with Mooren’s ulcer [16,24,25]. Despite the use of the systemic immunosuppressive therapy in all patients, additional AMT was necessary because of a persistent peripheral corneal ulceration or a progressive corneal thinning. These findings are in accordance with the results of Mondino and Spelsberg who report on a progression of Mooren’s ulceration under systemic immunosuppressive therapy in some cases [25,26].

It is well known that AMT is able to improve the corneal epithelialisation and to support the remission of inflammation, neovascularisation, and corneal scars in several corneal diseases. The mechanism of action of AMT is to induce the apoptosis in inflammatory cells, the release of protease inhibitors, and suppression of fibroblast proliferation [27]. The most important growth factors that promote wound healing, which have been isolated from the AM, are epidermal growth factor and keratocyte growth factor [28,29]. In addition, the AM produces basic fibroblast, hepatocyte and transforming growth factor (TGF). These growth factors together can stimulate epithelialisation, modulate proliferation, and induce differentiation of stromal fibroblasts [30].

The inhibition of TGF-β signal transduction in corneal fibroblasts explains AM’s anti-scarring effect [31]. In the initial phase after AMT, a significant reduction in inflammation is typical. The AM reduces expression of various growth factors and pro-inflammatory cytokines (e.g. interleukin 1a, IL -2, IL-8, interferon γ, tumor necrosis factor-β, basic fibroblast growth factor and platelet derived growth factor) [32]. Furthermore, the AM attracts and adheres to inflammatory cells infiltrating the ocular surface. In addition, the AM contains various forms of protease inhibitors [33]. This may explain some of its antiinflammatory properties. The reported immunomodulatory effect underlines the suggestion that AMT has a positive effect in healing Mooren’s ulcer [34].

Recently, a retrospective study of 18 eyes reported a stabilization of the visual acuity and rapid healing of the epithelial defect after single AMT in most cases [13]. Ngan and Chau reported in a rapid epithelialisation and a good visual outcome especially in patients with limited corneal ulceration. In patients with 360° ulceration, as was seen in most of our cases, the time of epithelialisation was up to one month and the visual outcome was poor. Another limitation of this study is the short follow up time (1.5 to 20 month). In addition, the authors did not ensure their diagnosis by assessing HLA-DQ2 and HLA-DR17(3).

The reported results of AMT combined with conjunctival autografting or lamellar keratoplasty are less convincing [14,15]. The authors claimed a positive effect of AMT in their studies but there are several limitations. Zhou and colleagues combined AMT with lamellar keratoplasty for patients with recurrent Mooren’s ulcer. They found a delayed recurrence in their cases but they did not differentiate the effect of lamellar keratoplasty from the AMT. In addition, they reported a short follow up time (12 to 29 month). Chen et al. reported on one case of initial successfully sealed corneoscleral perforation, a relapse after 2 months with infiltration along the conjuncival graft. After removing the conjuntival graft and a second AMT the leason was stable over 1 year. One case has a very limited value in a disease in which the natural course is very different and spontaneous remissions are described.

In contrast to those studies we report on cases of aggressive Mooren’s ulcer with large coneal ulcers in which AMT was not able to stabilize the processes of the disease. In most of our cases (6 of 7) we could identify HLA-DQ2 or HLA-DR17(3) which seems to be highly associated to Mooren’s ulcer and may be a prognostic factor. These findings emphasize our diagnosis of Mooren’s ulcer.

The anti-inflammatory potential of amniotic membrane may be useful in Mooren’s ulcer in some cases to support the systemic immunosuppressive therapy. The fact that 5 patients needed multiple AMT showed clearly that amniotic membrane is not able to stop the immunological process or even cure Mooren’s ulcer in aggressive processes.

## Conclusion

In conclusion, the aggressive highly inflammatory form of Mooren’s ulcer is difficult to treat and so far there is no optimal therapy for Mooren’s ulcer. The progression of the disease is hard to influence positively even under systemic immunosuppressive therapy and intensive local medication. Therefore, the main intention of therapy is to achieve a stable epithelialized corneal surface without the risk of perforation than to maintain the visual acuity. AMT is not able to cure severe forms of Mooren’s ulcer because the disease is based on an immunological process. However it supports the immunosuppressive therapy in acute situations like critical corneal thinning or persistent epithelial defects.

## Abbreviations

AM: Amniotic membrane; AMT: Amniotic membrane transplantation; HLA: Human leukocyte antigene.

## Competing interests

The authors declare that they have no competing interests.

## Authors’ contribution

MS participated in conception and design, data collection, data analysis and drafting the manuscript. HW participated in data collection, data analysis and critically revising the manuscript. K-PS performed the operations and participated in data analysis and critically revising the manuscript. DM performed the operations and participated in conception and design, data collection, data analysis and critically revising the manuscript. All authors read and approved the final manuscript.

## Pre-publication history

The pre-publication history for this paper can be accessed here:

http://www.biomedcentral.com/1471-2415/13/81/prepub
